# Assessment of Growth, Yield, and Nutrient Uptake of Mediterranean Tomato Landraces in Response to Salinity Stress

**DOI:** 10.3390/plants12203551

**Published:** 2023-10-12

**Authors:** Theodora Ntanasi, Ioannis Karavidas, Georgios Zioviris, Ioannis Ziogas, Melini Karaolani, Dimitrios Fortis, Miquel À. Conesa, Andrea Schubert, Dimitrios Savvas, Georgia Ntatsi

**Affiliations:** 1 Laboratory of Vegetable Production, Department of Crop Science, Agricultural University of Athens, Iera Odos 75, 11855 Athens, Greece; ntanasi@aua.gr (T.N.); karavidas@aua.gr (I.K.); geoziov95@gmail.com (G.Z.); ziogasg15@gmail.com (I.Z.); melini.karaolani@gmail.com (M.K.); fortisdim@gmail.com (D.F.); dsavvas@aua.gr (D.S.); 2 INAGEA-PlantMed, Departament de Biologia, Universitat de les Illes Balears, Carretera de Valldemossa km 7.5, E-07122 Palma, Illes Balears, Spain; ma.conesa@uib.es; 3 Plant Stress Lab, Department of Agriculture, Forestry and Food Sciences DISAFA, Turin University, 10095 Grugliasco, Italy; andrea.schubert@unito.it

**Keywords:** soilless culture, tomato, landraces, abiotic stress, growth, yield, nutrient concentration

## Abstract

Salinity is a major stress factor that compromises vegetable production in semi-arid climates such as the Mediterranean. The accumulation of salts in the soil can be attributed to limited water availability, which can be exacerbated by changes in rainfall patterns and rising temperatures. These factors can alter soil moisture levels and evaporation rates, ultimately leading to an increase in soil salinity, and, concomitantly, the extent to which crop yield is affected by salinity stress is considered cultivar-dependent. In contrast to tomato hybrids, tomato landraces often exhibit greater genetic diversity and resilience to environmental stresses, constituting valuable resources for breeding programs seeking to introduce new tolerance mechanisms. Therefore, in the present study, we investigated the effects of mild salinity stress on the growth, yield, and nutritional status of sixteen Mediterranean tomato landraces of all size types that had been pre-selected as salinity tolerant in previous screening trials. The experiment was carried out in the greenhouse facilities of the Laboratory of Vegetable Production at the Agricultural University of Athens. To induce salinity stress, plants were grown hydroponically and irrigated with a nutrient solution containing NaCl at a concentration that could maintain the NaCl level in the root zone at 30 mM, while the non-salt-treated plants were irrigated with a nutrient solution containing 0.5 mM NaCl. Various plant growth parameters, including dry matter content and fruit yield (measured by the number and weight of fruits per plant), were evaluated to assess the impact of salinity stress. In addition, the nutritional status of the plants was assessed by determining the concentrations of macro- and micronutrients in the leaves, roots, and fruit of the plants. The key results of this study reveal that cherry-type tomato landraces exhibit the highest tolerance to salinity stress, as the landraces ‘Cherry-INRAE (1)’, ‘Cherry-INRAE (3)’, and ‘Cherry-INRAE (4)’ did not experience a decrease in yield when exposed to salinity stress. However, larger landraces such as ‘de Ramellet’ also exhibit mechanisms conferring tolerance to salinity, as their yield was not compromised by the stress applied. The identified tolerant and resistant varieties could potentially be used in breeding programs to develop new varieties and hybrids that are better adapted to salinity-affected environments. The identification and utilization of tomato varieties that are adapted to salinity stress is an important strategy for promoting agriculture sustainability, particularly in semi-arid regions where salinity stress is a major challenge.

## 1. Introduction

In the Mediterranean basin, the high concentration of NaCl in irrigation water poses a major problem [1]. Globally, approximately 7% of soil is affected by salinity [2]. Salinity can be classified as primary salinity, which occurs due to environmental conditions such as reduced rainfall, wind, or natural processes like erosion, or secondary salinity, resulting from human activities such as excessive water and fertilizer usage [3]. Salinity induced by climate change refers to the excess accumulation of Na^+^ and/or Cl^−^ in the rhizosphere [4]. Ions can accumulate in water when the plant’s concentration is lower than that of the initial irrigation water [5]. Salt stress poses severe challenges for vegetable crops, particularly tomatoes, as it inhibits growth and biomass production by negatively impacting cell division and elongation [6]. Furthermore, it adversely affects the yield and tomato fruit size [7]. The reduced yield is a consequence of a decrease in the number of fruits, which, according to Cuartero and Fernandez-Muñoz (1998) [8], is associated with a reduction in flower production with increasing salinity. According to Gama et al. (2007) [9], the increased NaCl concentration in the roots leads to imbalances in different plant parts, especially for nutrients such as K, Ca, and Mg ions, for which assimilation problems are caused [10].

Tomato (*Solanum lycopersicum* L.) is the second most important crop worldwide in terms of production and consumption after potato, with cultivation covering an area of 5.03 Mha and a production of 180 Mt (FAO) [11]. In the Mediterranean basin, tomato is the predominant vegetable crop, both in outdoor and greenhouse cultivation [12]. A tomato is a plant characterized by a moderate resistance to salt stress [13,14], with variations in tolerance depending on the genotype [15]. Consequently, tomatoes can be successfully grown with an electrical conductivity (EC) of up to 2.5 dS/m without compromising yield [16]. According to Campos et al. (2006) [17], for each unit increase in salinity above 2.5 dS/m, there is an approximate 10% reduction in tomato yield.

Landraces, unlike modern cultivated varieties [18], offer the advantage of being able to adapt to adverse environments (e.g., salinity, drought, heat) without compromising yields [19,20]. Extensive research has been dedicated to identifying tolerance traits in tomato landraces that confer resilience to abiotic stresses such as drought and salinity [21,22]. Salt stress tolerant landraces or tomato genotypes exhibit the ability to alleviate the adverse effects of stress through mechanisms such as enhanced root development to overcome saline zones [23] or activation of biochemical and physiological processes that can enable ion and water homeostasis restoration [24].

Considering the aforementioned background, the present study aims to assess the impact of salinity on the growth, yield, and macro- and micronutrient concentration in the roots, leaves, and fruit of several Mediterranean tomato landraces. Their divergent response to the moderate salinity stress caused by a concentration of 30 mM NaCl in the rhizosphere is of great interest, as these traditional cultivars hold potential as valuable genetic resources for integration into breeding programs or as a promising tomato rootstock material in the near future. Additionally, they offer promising avenues for future research aimed at identifying genetic traits and mechanisms that contribute to improved fruit quality and enhanced resilience to salinity stress.

## 2. Results

### 2.1. Plant Growth Responses

**Figure 1:**
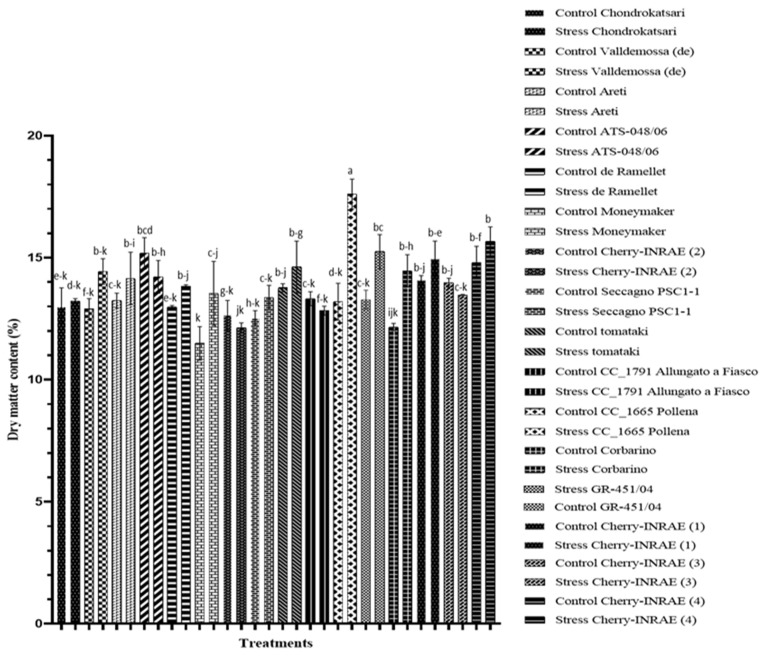
Impact of salinity stress on leaf dry matter content (%) of different tomato landraces. For each treatment, different letters at each bar indicate significant differences according to Duncan’s multiple range test (*p* < 0.05). Vertical bars indicate standard errors of means (*n* = 3).

The growth of tomato plants, as indicated by the dry matter content of the leaves (Figure 1), is significantly affected by salinity. Furthermore, this study reveals statistically significant differences among different cultivated genotypes and their interactions with salinity. According to Figure 1, the leaf dry matter content of the two cherry-type landraces, ‘CC_1665 Pollena’ and ‘Corbarino’, significantly increased by 25 and 16%, respectively, while at the same time the dry matter content of the leaves of the reference variety ‘Moneymaker’ increased by 15% when subjected to salinity stress. 

### 2.2. Yield 

Significant differences were observed among the different genotypes under salinity stress in terms of fruit production, as indicated by the mean fruit weight and the number of fruits per plant (Table 1). The impact of salinity on the number of fruits varied across different tomato genotypes. Notably, the mid-type landrace ‘Cherry-INRAE (2)’ and the cherry-type landraces ‘Corbarino’, ‘Cherry-INRAE (1)’, and ‘Cherry-INRAE (3)’ exhibit a reduction in fruit number when grown in a nutrient solution supplemented with 30 mM NaCl. Specifically, the above-mentioned genotypes produced approximately 19, 18, 13, and 21 fruits less than the control, respectively, when subjected to salinity stress. When considering mean fruit weight, the two beef-steak landraces, ‘Chondrokatsari’ and ‘Areti’, were the ones that, under stress conditions, had significantly reduced their mean fruit weight by 23 and 14%, respectively, compared to normal growth conditions.

**Figure 2:**
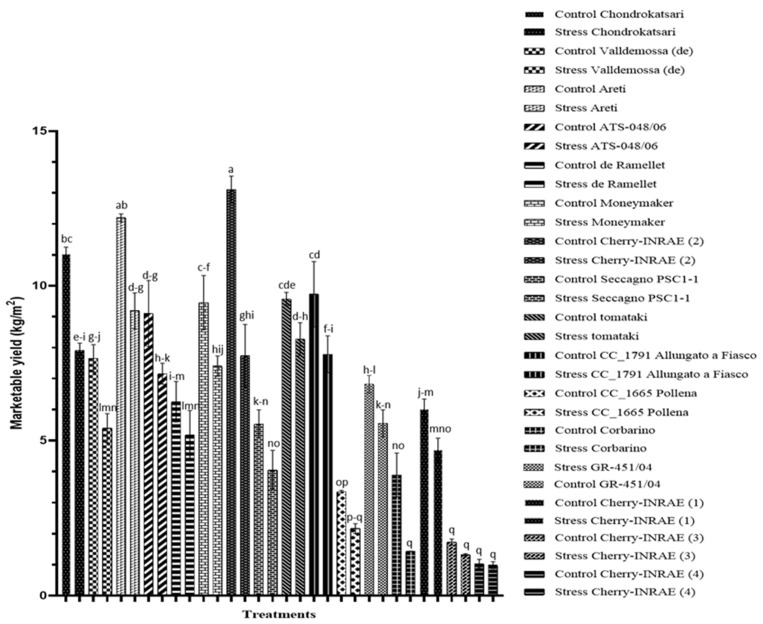
Impact of cultivation under saline conditions on the marketable yield (kgm^−2^) of different tomato landraces. For each landrace and treatment, different letters at each bar indicate significant differences according to Duncan’s multiple range test (*p* < 0.05). Vertical bars indicate standard errors of means (*n* = 3).

As illustrated in Figure 2, 30 mM of NaCl in the root environment of tomatoes differentially impacted the genotypes under study, with some showing a decrease in marketable yield while others remained unaffected. Among the genotypes evaluated, approximately half of them exhibit a decrease in marketable yield under stress conditions. Specifically, all four beef-steak landraces ‘Chondrokatsari’, ‘Valldemossa (de)’, ‘Areti’, and ‘ATS-048/06’ displayed significant reductions in marketable yield, with decreases of 28, 30, 25, and 21%, respectively, under the stress applied. In the mid-type ‘Moneymaker’ and ‘Cherry-INRAE (2)’, marketable yield was reduced by 22 and 41%, respectively, under stress conditions. Among the cherry-type landraces, ‘CC_1791 Allungato a Fiasco’ and ‘Corbarino’ exhibit significant reductions of 20 and 63%, respectively, in marketable yield when grown under salinity stress.

### 2.3. Concentration of Macro- and Micronutrients in Roots

The effect of salinity stress, the different genotypes, and their interaction on the concentrations of macronutrients (K, Na, Ca, and Mg) in plant roots was found to be significant (Table 2). Specifically, under salinity stress, the cherry-type landrace ‘GR 451/04’ exhibits a 50% reduction in K concentration in the roots compared to plants grown under normal conditions. The cherry-type landrace ‘CC_1665 Pollena’ and the mid-type reference variety ‘Moneymaker’ also show significant reductions of 43%, and the large-fruited ‘de Ramellet’ had a reduction of 37% in K concentration compared to those plants grown under normal conditions. 

Regarding the K/Na ratio in the roots, it is observed that of the large-fruited landraces, only the ‘de Ramellet’ does not show a difference under salinity conditions, while among the cherry-type ‘CC_1665 Pollena’, ‘Cherry-INRAE (1)’, ‘Cherry-INRAE (3)’, and ‘Cherry-INRAE (4)’ are the ones where the K/Na ratio did not show a significant difference between the two treatments (0.5 and 30 mM NaCl).

Regarding Ca concentration, the mid-type landrace ‘de Ramellet’ shows a significant decrease of 50% in the roots under high NaCl concentration in the nutrient solution. However, the cherry-type varieties exhibit a lesser decrease in Ca concentration under salt stress. In particular, ‘CC_1665 Pollena’ shows an increase of 13%, and ‘CC_1791 Allungato a Fiasco’ shows an increase of 22% in Ca concentration in the roots compared to the control.

The beefsteak landrace ‘Chondrokatsari’ displayed a significant decrease of 55% in Mg concentration in the roots under salinity stress conditions. In contrast, no significant decrease in root Mg concentration of the cherry-type landraces ‘CC_1791 Allungato a Fiasco’, ‘CC_1665 Pollena’ and ‘Corbarino’ was found.

Regarding the salinity factor, the addition of NaCl affected the Na concentration in the roots, which increased under stress conditions. The cherry-type landraces ‘CC_1791 Allungato a Fiasco’ and ‘Corbarino’ exhibit the highest percentage of sodium in the roots. On the other hand, the large-type landrace ‘de Ramellet’ and the cherry-type ‘Cherry-INRAE (4)’ had the lowest Na concentration in their roots. No significant difference in the Na root concentration was observed between the two treatments (0.5 and 30 mM NaCl) for the three other cherry-type landraces ‘Cherry-INRAE (1)’, ‘Cherry-INRAE (3)’, and ‘CC_1665 Pollena’.

Among the four trace elements studied (Fe, Cu, Mn, and Zn), three of them (Fe, Cu, and Mn) show statistically significant differences in response to stress in the different landraces, while zinc (Zn) did not exhibit significant changes. Regarding the concentration of Fe in plant roots, the variety ‘Moneymaker’ used as a reference shows the greatest reduction of about 67% under stress conditions. On the other hand, the cherry-type landrace ‘Cherry-INRAE (1)’ displayed a slight increase in Fe concentration with a tendency towards increasing levels under salt stress.

For Cu concentration, the landrace ‘de Ramellet’ exhibits the greatest reduction of 37% under salinity conditions. In contrast, the cherry-type landrace ‘Cherry-INRAE (1)’ did not show any significant change in Cu concentration in the roots when exposed to salinity stress.

The concentration of Mn in the roots of plants of the beef-steak landrace ‘Chondrokatsari’ shows the greatest decrease compared to the other varieties, with a reduction of about 41%. On the other hand, the cherry-type landraces ‘Cherry-INRAE (4)’, ‘Cherry-INRAE (1)’, and the mid-type ‘Seccagno PSC1-1’ did not display any significant changes in Mn concentration in their roots when subjected to salinity stress. As shown in Table 3, salinity stress significantly reduced the concentrations of Zn in the roots of tomato plants by about 9%. Furthermore, the different cultivars had statistically significant differences in the concentration of Zn in their roots. Specifically, the Zn concentration of the cherry-type landrace ‘tomataki’ was close to the highest concentration of the cherry-type landrace ‘Cherry-INRAE (1)’ (122,42 μg/g DW).

According to Table 4, salt stress significantly increased the concentration of potassium (K) by 18% while the concentration of magnesium (Mg) remained unaffected.

### 2.4. Concentration of Macro- and Micronutrients in Leaves

The different cultivars show significant differences for leaf K and Mg contents. Among the tested genotypes, the mid-type landrace ‘Seccagno PSC1-1’ and the cherry-type landraces ‘GR-451/04’, ‘Corbarino’, and ‘Cherry-INRAE (4)’ displayed the highest K concentration, while the two beefsteaks ‘ATS-048/06’, ‘Valldemossa (de)’, and a cherry-type landrace ‘tomataki’ had the lowest K concentrations, which were approximately half of the concentration observed in the genotypes with the highest K levels. The genotypes in which the leaf K/Na ratio did not change under salt stress conditions were the cherry-type landraces ‘CC_1665 Pollena’, ‘Cherry-Inrae (3)’, and ‘Cherry-Inrae (4)’.

Among the studied genotypes, the beef-steak landrace ‘Valldemossa (de)’ and the two cherry-type landraces ‘Cherry-INRAE (4)’ and ‘Corbarino’ exhibit the highest Mg concentration, reaching approximately 10 mg/g dry weight (DW).

In terms of leaf Na content, all cultivars grown under salt stress displayed increased concentrations in their leaves. The beef-steak ‘Chondrokatsari’ was the one with the highest increase under stress conditions, approximately 88%, while the smallest increase of 61% was observed in the beef-steak landrace ‘ATS-048/06’. No significant difference in the Na leaf concentration was observed between the two treatments (0.5 and 30 mM NaCl) for the landraces ‘Cherry-INRAE (3)’, ‘Cherry-INRAE (4)’, ‘CC_1665 Pollena’, and ‘GR_451/04’. As for Ca, the reference cultivar ‘Moneymaker’ demonstrates the largest decrease of about 30% in leaf concentration under salt stress.

A significant interaction among the treatments (salinity × genotype) was evident for all micronutrients in the leaves (Table 5). For Fe, the cherry-type landrace ‘GR-451/04’ increased its leaf Fe concentration by 49% under stress conditions, while the beefsteak landrace ‘Chondrokatsari’ shows a decrease of 35% in Fe concentration.

In terms of Cu, the beef-steak landrace ‘Areti’ increased its leaf concentration under salinity stress conditions, while the cherry-type variety ‘Cherry-INRAE (1)’ exhibits a 66% decrease. Regarding Mn concentration in the leaves, a decrease was observed in the beef-steak and cherry-type varieties under salinity stress conditions. The decrease ranged from 60% in ‘de Ramellet’, 32% in ‘Chondrokatsari’, 26% in ‘Cherry-INRAE (2)’, and 39% in ‘Moneymaker’, to 60% and 46% in the cherry-type ‘tomataki’. Finally, for Zn, some beef-steak and cherry-type varieties show a decrease in leaf concentration under saline conditions, while others exhibit significantly higher concentrations. Specifically, the large-type ‘de Ramellet’, ‘Valldemossa (de)’, ‘ATS-048/06’, and ‘Areti’ and a cherry-type variety ‘GR-451/04’ were the ones that show a decrease of the micronutrient in their leaves when grown under salinity conditions, while the mid-type ‘Seccagno PSC1-1’ and the cherry-type Cherry-INRAE (4)’ were the ones that show a significantly higher concentration of Zn under stress conditions. The cherry-type landraces ‘CC_1791 Allungato a Fiasco’ and CC_1665 Pollena’ did not show a significant difference in any of the measured micronutrients between the salinity treatments.

### 2.5. Concentration of Macro- and Micronutrients in Fruit

As shown in Table 6, a significant interaction among the treatments was found for all macronutrients in the tomato fruit. Specifically, the beef-steak landraces ‘de Ramellet’ and ‘Cherry-INRAE (2)’ and the two cherry-type landraces ‘CC_1665 Pollena’ and ‘tomataki’ exhibit a reduction in K concentration under stress conditions by 15%, 19%, 19%, and 16%, respectively. Τhe K/Na ratio in fruit did not have any statistically significant difference for the cultivation of the different genotypes under saline stress. A significant decrease in the Ca concentration in the fruits was observed in the beef-steak landrace ‘Areti’ and in the cherry-type landrace ‘GR-451/04’ cultivated under increased NaCl in the nutrient solution. A corresponding decrease was also observed in the Mg concentration in the fruit of large-type landraces ‘Valldemossa (de)’ and ‘de Ramellet’ as well as the cherry-type ‘CC_1665 Pollena’, ‘Corbarino’, and ‘Cherry-INRAE (1)’. On the other hand, Na concentration increased in almost all varieties under salinity stress conditions. However, variations among the varieties were identified. The landrace ‘CC_1665 Pollena’ shows the same Na fruit concentration in both treatments, indicating a limited response to salinity. The landraces ‘Cherry-INRAE (1)’, ‘Cherry-INRAE (4)’, and ‘Corbarino’ exhibit the lowest increase of this element in the fruit compared to all other varieties in the study.

The interaction between salinity and the different cultivars shows statistically significant differences for all measured micronutrients (Table 7). Among the landraces, the large-type variety ‘de Ramellet’ was the one with the largest decrease in all fruit trace element concentrations under stress conditions. Specifically, the fruit of this variety shows a reduction of approximately 40% in Fe, 50% in Cu, 30% in Mn, and 14% in Zn. The cherry-type landrace ‘GR-451/04’ also displayed significant reductions in Fe, Mn, and Zn fruit concentrations, with decreases of 43%, 25%, and 18%, respectively. Interestingly, this variety shows a significant increase of 40% in Cu content in the fruit of stressed plants. Finally, the beef-steak landrace ‘Areti’ shows reductions of 37% in Fe concentration and 15% in Mn concentration in the stressed fruit. 

### 2.6. Sodium Content (%)

**Figure 3:**
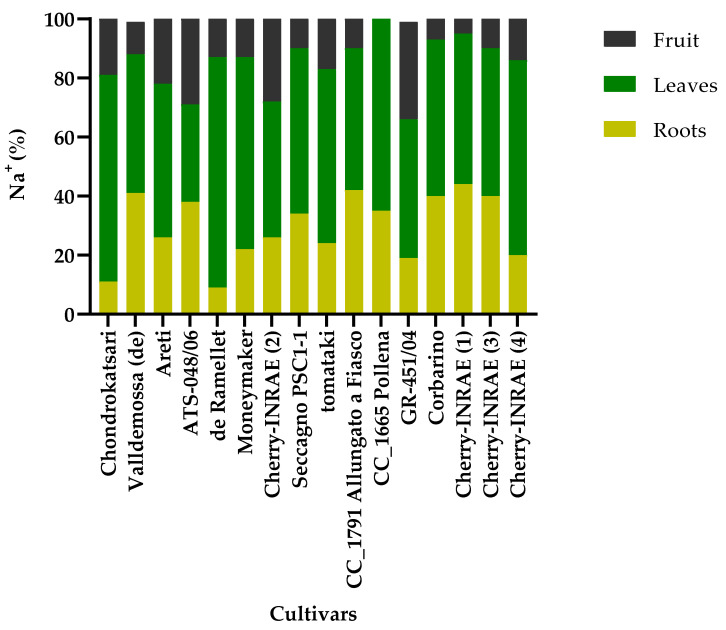
Na content (%) in the different plant parts (roots, leaves, and fruits).

As shown in Figure 3, the distribution of sodium was different in the plant parts determined in the present study for the different genotypes. In a general context, the highest Na content was found in the leaves, then in the roots, and the lowest Na content was found in the fruit. However, this distribution was genotype-dependent. It is observed that the large-sized landraces ‘Chondrokatsari’ and ‘de Ramellet’ have a similar distribution of Na in the parts of the plant. However, there are also genotypes such as the large-sized landrace ‘Areti’ and the cherry-type ‘GR-451/04’ in which the Na content appears to be almost equally distributed in leaves, roots, and fruits. Conversely, in the cherry-type landraces, only a small percentage was found in the fruit, while the rest was distributed in the leaves and the roots. 

## 3. Discussion

High salinity has a detrimental impact on tomato plant biomass [25]. Romero-Aranda et al. (2001) [26] explained this phenomenon as a decrease in water potential in leaves due to the presence of high salinity in their rhizosphere, which adversely affects various plant processes. Additionally, under salt stress, the excessive accumulation of Na and the concomitant nutrient imbalances in plants further impede their growth [27]. In the present study, the exposure of tomatoes to a NaCl concentration of 30 mM in the nutrient solution retained in the root zone (root solution) increased the dry matter content of the leaves. This finding aligns with the research conducted by Douglas McCall and Aušra Brazaityte (1997) [28], where increasing EC resulted in increased shoot dry matter content. According to Adams et al. (1990) [29], a negative correlation exists between NaCl concentration and leaf area in tomato plants, as increased NaCl levels led to a reduction in leaf size [30,31]. Nonetheless, in the present study, notable variations among the landraces were found, indicating the divergent tolerance of different tomato landraces to salinity stress [32,33]. Among the landraces examined, ‘CC_1665 Pollena’, ‘Corbarino’, and ‘GR-451/04’ exhibit a statistically significant increase in dry matter content under saline conditions (Figure 1). However, it is worth noting that only ‘Corbarino’ displayed a significant decrease in marketable yield (Figure 2).

To evaluate the effect of salinity on the different tomato landraces under study, fruit yield was assessed at a moderate concentration of NaCl (30 mM) in the root zone. According to Saranga et al. (1991) [34], a yield reduction of approximately 10% occurs for each 1 dSm^−1^ increase above the threshold of 2.5 dSm^−1^, which signifies a decline in production. Furthermore, Rodríguez-Ortega et al. (2019) [35] reported a reduction of 35% and 58% in marketable yield in soilless tomato crops exposed to 40 mM and 80 mM NaCl, respectively, compared to the control. However, the response of tomato genotypes to salt stress exhibits notable variation. Caro et al. (1991) [36] demonstrated that cherry-type cultivars display higher tolerance to saline stress compared to those with normal-sized fruits. In our study, eight of the tested genotypes did not show a statistically significant decrease in marketable yield. More specifically, the large-sized ‘de Ramellet’ and the mid-type ‘Seccagno PSC1-1’ with reduction percentages of 17% and 26%, respectively, were the two genotypes that did not show a statistically significant difference in their yield under salinity stress conditions. In addition, a non-significant reduction in marketable yield was observed in six cherry-type landraces. These landraces were ‘CC-1665 Pollena’, ‘GR-451/04’, ‘tomataki’, ‘Cherry-INRAE (1)’, ‘Cherry-INRAE (3)’, and ‘Cherry-INRAE (4)’ with 35%, 18%, 14%, 22%, 24%, and 5% reduction, respectively (Figure 2).

In the study conducted by Liu et al. (2014) [37], it was found that the addition of 50 mM of nutrient solution resulted in a 21% reduction in the number of fruit per plant in the tomato variety (TA19) compared to the control. Similarly, Magán et al. (2008) [7] reported that the reduced yield in tomato production was attributed to a decrease in the number of fruits per plant with increasing salinity. Consistent with these findings, the present study reveals a significant decrease in fruit number per plant under moderate salinity conditions of 30 mM in the root zone, amounting to approximately 14%. The cherry-type landraces ‘Corbarino’, ‘Cherry-INRAE (1)’, ‘Cherry-INRAE (3)’, and ‘Cherry-INRAE (4)’ were the landraces in which a statistically significant reduction of 18, 14, 19, and 21%, respectively, was observed. In contrast, the other genotypes did not change their fruit number under salinity stress conditions (Table 1). This decline in fruit number can be attributed to the disruption of the plant’s physiological functions caused by osmotic stress and the consequent imbalance of nutrients resulting from the increased concentration of salt [38]. Therefore, our results indicate that the decline in production was primarily driven by a decrease in the average fresh fruit weight and the number of fruits per plant [39], and this effect was genotype-dependent. Therefore, landraces that, under saline stress conditions, did not reduce their yield can be characterized as tolerant to this salinity level (30 mM NaCl), as according to Maas and Hoffman (1977) [16], crops are considered resistant to the level of salinity that does not affect their yield.

Τhe addition of NaCl to the nutrient solution leads to an increase in the Na concentration within the plant tissues [40]. Sodium accumulation varies across different plant parts [24], reflecting its distinct distribution within plant organs. According to Babu et al. (2012) [41], the Na content of tomato leaves increases more significantly than in fruits when cultivated under high NaCl concentrations. Similarly, in the present study, the Na content in the leaves of the stressed plants was approximately five times higher compared to the control plant, while the increase in fruits was twice as high and in roots was three times higher than in the control plant (Figure 3). Alfocea et al. (1993) [42] reported that different genotypes of *L*. *esculentum* exhibit diverse responses to salinity, either by replacing K with Na or through K selectivity. In addition, the K/Na ratio determined in the present study is considered a selection factor for resistant cultivated species to salinity [43]. The ability of plants to exclude salt is one of the mechanisms of salt tolerance [44]. ‘Cherry-INRAE (1)’, ‘Cherry-INRAE (3)’, ‘Cherry-INRAE (4)’, and ‘de Ramellet’ landraces can be considered tolerant to moderate salinity as these genotypes did not show a significant difference in Na concentration or K/Na in the roots of stressed plants compared to the control (Table 6). Additionally, these cultivars did not exhibit a significant difference in yield under the stress applied. 

Potassium is a crucial macronutrient for tomato plants and plays a significant role in achieving high fruit quality [45,46]. However, under increased concentrations of NaCl, a nutrient imbalance occurs due to the substitution of K+ by n plants by Na+ in plant tissues. In the present study, a reduction in K concentration under salinity stress conditions was observed, with roots showing a decrease of 25% and leaves exhibiting a decrease of 20%. Specifically, the genotypes that show a significant reduction of K in their roots were the large-sized landraces ‘Areti’ at a 29% and 38% reduction, respectively, the mid-type variety ‘Moneymaker’ at 43%, and the cherry-type landraces ‘CC_1665 Pollena’ and ‘GR-451/04’ with a reduction at 43% and 53%. Regarding leaf K concentration, there was no statistically significant interaction for the genotypes under salinity conditions. This finding is consistent with the study conducted by Yan Li et al. (2009) [47], who reported a greater decrease in K ions in roots compared to leaves. Furthermore, a 5% decrease in Κ concentration was observed in the fruit of the present study, in agreement with the study of Babu et al. (2012) [41], where the concentration of potassium ions in the fruit decreased with increasing NaCl concentration. The large-type landrace ‘de Ramellet’ and the mid-type ‘Cherry-INRAE (2)’ under salinity stress conditions show a 16% and 19% reduction of K in their fruits, respectively. At the same time, the cherry-type landraces ‘CC_1665Pollena’ and ‘tomataki’ show a decrease of 19% and 16%, respectively, in K under stress conditions. According to Adams and Ho (1995) [48], the competition of these two macronutrients leads to reduced uptake of potassium by plants under salinity conditions, and this reduction is more strongly related to decreased water uptake. Among the landraces studied, the large-fruited landrace ‘de Ramellet’ and the cherry-type ‘CC_1665 Pollena’ exhibit the most significant reductions in K concentration in both roots and fruit when grown under salinity conditions. Similarly, the K/Na ratio decreased in the leaves, fruits, and roots of tomatoes with the addition of NaCl to the nutrient solution. This observation aligns with the finding of Taffouo et al. (2010) [49], who observed a decrease in the K/Na ratio in the leaves and roots of tomato cultivars grown under salinity stress. In a broader context, the small-sized tomatoes in this study were those with the highest K/Na ratios, indicating greater salinity tolerance. This observation is consistent with several studies [50,51,52,53] that highlight the ability of plants with high K/Na in their tissues to exhibit salinity tolerance.

Under high NaCl conditions, tomato plants exhibit reduced Ca uptake [54], which is not solely due to competition with Na but also attributed to a decrease in transpiration rate caused by salinity stress [8]. In the present study, salinity negatively affected the Ca concentrations in the roots, leaves, and fruit of tomato plants. Similar findings have been reported in other studies, where plants subjected to salinity stress exhibited a decrease in Ca concentration in leaves [55] and fruit [56]. This reduction in Ca concentration is associated with increased hydraulic resistance caused by high NaCl levels, resulting in reduced water and Ca transport. The roots of the large-type landraces ‘de Ramellet’, ‘Chondrokatsari’, and ‘Valldemossa (de)’ show a significant decrease of 50% in Ca concentration. In contrast, the other landraces did not display a significant reduction in the concentration of this ion. The retention of Ca in roots can be attributed to either a decrease in Na uptake and an increase in K uptake [44,57] or the maintenance of K concentration [58], which contributes to the proper growth and the maintenance of adequate K levels in plants. 

The concentration of Mg in the leaves of the cultivated tomato landraces was not significantly affected by salinity. This finding aligns with previous studies that reported no significant impact of NaCl on Mg content in tomato leaves [59]. On the contrary, a significant decrease in Mg concentration was observed in the roots of plants subjected to salinity, which is consistent with the findings of Li et al. [60] and Yunus and Zari [59]. In particular, the large-type landraces ‘de Ramellet’, ‘Chondrokatsari’, and ‘Valldemossa (de)’ exhibit the highest decrease in Mg content in their roots. Furthermore, under high NaCl, a decrease in Mg concentration in tomato fruits was observed, although to a lesser extent (approximately 5%) compared to the leaves. Among the landraces, ’de Ramellet’ and ‘Valldemossa (de)’ also show a decrease in fruit Mg content under saline conditions.

The concentration of micronutrients (Fe, Cu, Mn, and Zn) in tomato tissues has been found to decrease with increasing NaCl [61]. Consistent with previous studies on tomato cultivars [62], the present study also observed a decrease in fruit micronutrient concentrations under salinity conditions. Nouck et al. [63] similarly found that NaCl addition in the nutrient solution reduced the concentrations of Fe, Cu, Mn, and Zn in the roots of different tomato cultivars, confirming the findings of the present study. Variations in salinity tolerance and micronutrient accumulation were also observed among different tomato varieties, as reported by Nouck et al. [63]. In terms of leaf responses to salinity stress, an increase in Fe concentration was observed, in line with the findings of García Fuentes et al. [64], while the Mn concentration of tomato leaves decreased, as also reported by Alam et al. [65]. The Cu concentration in leaves did not show significant differences, consistent with the study of El-Fouly et al. [66]. Likewise, no differences were observed in leaf Zn concentration, consistent with the findings of García Fuentes et al. [64] and Kowalska and Smolen [67]. In the present study, variations in the micronutrient concentrations (Fe, Cu, Mn, and Zn) among different landraces under salt stress were observed. Notably, the variety ‘CC_1791 Allungato a Fiasco’ did not show any changes in micronutrient concentrations in response to salinity. Furthermore, for the landraces ‘Cherry-INRAE (4)’ and ‘Seccagno PSC1-1’, the increase in NaCl either had no effect or increased the concentration of Fe and Zn, but only in their leaves and not in other plant parts analyzed. 

## 4. Materials and Methods

### 4.1. Experimental Design and Plant Material

The experiment was carried out at the greenhouse facilities of the Laboratory of Vegetable Production of the Agricultural University of Athens (AUA), located at coordinates 37°59′2″ N and 23°42′19″ E. Fifteen different Mediterranean tomato landraces and one tomato variety, ‘Moneymaker’, as a reference, were cultivated in an open soilless culture system by applying two different concentrations (0.5 and 30 mM) of NaCl in the nutrient solution. The name and the seed source of the cultivated tomatoes, including the fifteen landraces and the variety ‘Moneymaker’, are provided in Table 8. The experiment was laid out as a randomized complete block design (RCBD) by applying two different concentrations of NaCl (0.5 and 30 mM) in the nutrient solution retained in the root zone (root solution). 

### 4.2. Growth Conditions and Cultivation Practices 

On 30 January 2021, the tomato seeds underwent a disinfection process using a 15% *v*/*w* trisodium phosphate (Na_3_PO_4_) solution. Following disinfection, the tomato seeds were placed in a temperature-controlled (25 °C) incubation chamber for germination. After three days, the germinated seeds were transplanted into sowing trays using turf as a substrate. On March 10, when the seedlings had developed four or five true leaves, they were transplanted into perlite bags (33 L), and the cultivation was performed in an open hydroponic system. Each treatment was replicated three times (3 perlite bags/treatment). Each perlite bag accommodated 3 plants of the same variety and was fed with nutrient solution (NS) from a ‘supply-tank’ via a pump and a drip irrigation system. The bottom of each bag was slit to allow the free drainage of NS supplied in excess of the plants’ demand. The mean temperature during the whole cultivation period was 21 °C (day) and 17 °C (night), respectively.

### 4.3. Nutrient Solution Formula

To calculate the nutrient solution (NS) applied to tomato plants, the NUTRISENSE online tool (accessible at https://nutrisense.online) was used. The concentration of nutrients in the supplied nutrient solution varied depending on the treatment. Half of the plants were grown using a nutrient solution (NS) containing 30 mM NaCl (salinity treatment), while the other half were grown with a nutrient solution containing 0.5 mM NaCl (control treatment). The macro- and micronutrient concentrations in the nutrient solution for each treatment and developmental stage can be found in Table 9. The pH in the nutrient solution was adjusted to 5.6 daily by adding appropriate amounts of 1 N HNO_3_ stock solution. 

### 4.4. Application of Salt Stress in Tomato Experiments Conducted in an Open Hydroponic System

To apply salt stress at this level, we followed the procedure described below: 

A starter nutrient solution was prepared with a final concentration of 30 mmol L^−1^ (including the NaCl of the irrigation water) and an EC of 6.4–6.5 dS m^−1^. The starter NS was used to moisten the substrate in substrate-grown crops up to saturation, and then action was taken by silting the bottom of the substrate to enable free drainage, thereby reducing the moisture status to container capacity in the perlite. 

The NaCl concentration was calculated in the NS used to fertigate the plants after transplanting (C_t_) using Equation (1):

C_t_ = aC_d_ + (1 − α) C_u_ (1)

The target drainage fraction (a) in (1) was substituted by a suitable value (normally ranging from 0.1 to 0.35). Furthermore, C_d_ in (1) was replaced by 30 mmol L^−1^ since this is the target NaCl concentration in the root zone. The uptake concentration of Na (C_u_) in (1) was calculated using the relationship suggested by Carmassi et al. (2005) [68] for tomatoes:

C_u_ = 0.18C_r_ (2)

Equation (2) enables the calculation of the actual Na UC (C_u_) in tomato crops as a relationship of the actual Na^+^ concentration in the root environment (C_r_). Substituting C_r_ by 30 mmol L^−1^ (the target Na^+^ concentration in the root zone) in (2) renders a C_u_ of 5.4 mmol L^−1^. Replacing C_u_ with 5.4 mmol L^−1^ in (1) and with 0.3 (a standard drainage fraction) renders a C_t_ of 12.8 mmol L^−1^.

A standard NS for open soilless cultivation of tomato with an initial EC of 2.6–2.8 dS m^−1^ was prepared after the addition of the fertilizers and NaCl at a concentration of 12.8 mmol L^−1^. The addition of NaCl at this concentration is anticipated to increase the EC by 1.47 dS m^−1^, thereby rendering a final EC of about 4.2 dS m^−1^. This NS was used to supply the tomato plants in the salt stress treatment after their transplanting. 

Sodium concentration was measured weekly in the drainage solution to control the NaCl level. If the measured Na concentration was substantially different than 30 mmol L^−1^, C_ra_ was replaced by the measured value in (3) to estimate the actual C_u_. Then use (1) again, as described in step 2, to calculate a new value for C_t_. At the first measurement of the Na concentration in the drainage solution, C_rp_ by 30 mmol L^−1^ was replaced, while in all subsequent measurements, the Na concentration was measured in the previous week as C_rp_ to adjust C_t_. Subsequently, we repeated step 3 using the new C_t_ in place of 12.8 mmol L^−1^.

C_u_ = [2dV_s_ C_t_ − 2V_r_ (C_ra_ − C_rp_) − daVs (C_ra_ + C_rp_)]/(2d(1 − α)V_s_) (3)

### 4.5. Sampling and Measurements

At the end of the experiment, all tomato plants per treatment replication were sampled. From every plant, the fresh weight of the 3rd, 4th, and 5th leaf from the top was weighed. Its root was cleaned from perlite and rinsed with water. In addition, during the harvest, fruit samples were taken and their fresh weight was determined. The roots, leaves, and fruit were placed for drying at 65 °C to a constant weight. 

### 4.6. Growth Parameter: Dry Matter Content

The measurements of fresh weight and dry weight after drying at 65 °C were used to determine the dry matter content. Leaf dry matter content (%) was calculated using the relationship:

Dry matter content of leaf (%) = [Dry weight of leaf (g)/Fresh weight of leaf (g)] × 100

### 4.7. Yield

The harvest period started on 11 May 2021, and ended on 28 June 2021, and the fruits were harvested twice per week when they reached their commercial ripe stage. The total fruit number per plant, the total fruit fresh weight per plant (g plant^−1^), and the mean fresh fruit weight (g) were recorded. Marketable yield was classified as fruit without cracking, blossom-end rot (BER), blotchy ripening, or deformations.

### 4.8. Nutrient Concentrations

After drying, all samples were crushed in a mill MF 10 Basic Micro Fine Grinder (IKA Werke, Staufen, Germany). This powder was placed in porcelain capsules and placed for dry ashing in a muffle furnace at 550 for 8 h. The ash was dissolved in 0.5 N HCl. This extract was used for the determination of K and Na (flame photometer method, Sherwood Model 410, Cambridge, UK), and the concentration of Ca, Mg, Zn, Fe, Cu, and Mn was also determined using an atomic absorption spectrophotometer (AA-7000, Shimadzu Co., Tokyo, Japan).

### 4.9. Statistical Analysis

In the current study, a two-way ANOVA analysis was applied to identify the main effects of the NaCl stress on growth parameters, marketable yield, and nutrient composition of plant tissue of the different tomato genotypes. The data were statistically evaluated by applying ANOVA using the STATISTICA software package, version 12.0 for Windows. When either salinity stress at the different landraces had a significant impact on a measured parameter, the means within each factor were separated using the Duncan’s Multiple Range test (*p* ≤ 5%).

## 5. Conclusions

Based on this study, it has been observed that different tomato varieties exhibit varying responses to salt stress. In general, cherry-type varieties tend to display higher tolerance to saline environments, which can be attributed to the K/Na ratio, an important determinant of a plant’s tolerance to salinity. However, it is worth noting that there are also large-sized varieties that possess specific mechanisms that confer resistance to such stress. Among the tested varieties, the cherry-type landraces ‘Cherry-INRAE (1)’, ‘Cherry-INRAE (3)’, ‘Cherry-INRAE (4)’ and the large-fruited ‘de Ramellet’ demonstrate the highest tolerance to salinity in terms of both yield and growth characteristics, as well as nutrient concentration in the different plant parts. This is because the concentration of Na in the roots of these varieties remains unaffected, and their productivity remains intact under salt stress. This suggests that the above landraces could potentially be used as rootstocks in salinity-stressed areas. Moreover, their tolerance to moderate salinity stress constitutes them suitable candidates for breeding programs. By leveraging the characteristics of these varieties, it is possible to enhance tomato cultivation in challenging salinity conditions and promote the development of more resilient crop varieties.

## Figures and Tables

**Table 1:** Impact of cultivation under saline conditions on the number of fruits per plant and the mean fruit weight (MFW) of different tomato landraces. In the table, 0.5 mM NaCl refers to control (without NaCl addition), and “30 Mm NaCl” denotes salinity stress.

Marketable Yield			
Salinity Stress	Variety	No Fruit Plant^−1^	MFW (g)
Interaction			
0.5 mM NaCl	Chondrokatsari	8.22 m	448.72 a
	Valldemossa (de)	10.11 lm	252.66 c
	Areti	20.89 kl	195.27 d
	ATS-048/06	24.11 jk	125.99 f
	de Ramellet	19.00 klm	110.43 fg
	Moneymaker	33.44 ij	94.21 gh
	Cherry-INRAE (2)	57.33 fg	78.85 hij
	Seccagno PSC1-1	25.67 jk	72.16 h–k
	tomataki	59.33 d–g	53.94 i–m
	CC_1791 Allungato a Fiasco	58.02 efg	55.61 i–l
	CC_1665 Pollena	23.89 jk	46.88 k–n
	GR-451/04	66.56 c–f	34.28 l–p
	Corbarino	39.22 hi	32.50 l–p
	Cherry-INRAE (1)	83.78 a	23.96 m–p
	Cherry-INRAE (3)	69.22 b–e	8.32 op
	Cherry-INRAE (4)	77.22 abc	4.44 p
30 mM NaCl	Chondrokatsari	7.78 m	343.65 b
	Valldemossa (de)	8.00 m	231.00 c
	Areti	18.22 klm	168.15 e
	ATS-048/06	21.44 kl	111.80 fg
	de Ramellet	18.56 klm	94.81 gh
	Moneymaker	30.33 ijk	81.48 hi
	Cherry-INRAE (2)	38.22 hi	67.13 h–k
	Seccagno PSC1-1	18.83 klm	73.25 h–k
	tomataki	56.00 fg	49.17 j–n
	CC_1791 Allungato a Fiasco	55.78 fg	46.45 k–n
	CC_1665 Pollena	19.56 klm	36.92 l–o
	GR-451/04	63.33 def	29.41 l–p
	Corbarino	20.89 kl	23.59 m–p
	Cherry-INRAE (1)	69.89 bcd	22.37 nop
	Cherry-INRAE (3)	47.89 gh	9.51 op
	Cherry-INRAE (4)	79.89 ab	4.11 p
Statistical significance			
Salinity Stress		***	***
Variety		***	***
Salinity Stress × Variety		**	***

Mean values (*n* = 3) followed by different letters within the same column indicate significant differences according to Duncan’s multiple range test (*p* < 0.05). *** and ** are significant at *p* < 0.001 and *p* < 0.01, respectively (Table S1).

**Table 2:** Impact of cultivation under saline conditions on the concentration of macronutrients (K, Na, Ca, Mg, and K/Na) in the roots of different tomato landraces. In the table, 0.5 mM NaCl refers to control (without NaCl addition), and “30 Mm NaCl” denotes salinity stress.

Roots						
Salinity Stress	Variety	K (mg/g)	Na (mg/g)	K/Na	Ca (mg/g)	Mg (mg/g)
Interaction						
0.5 mM NaCl	Chondrokatsari	29.33 bcd	5.9 e–i	5.41 e–h	5.62 a–d	2.78 a
	Valldemossa (de)	29 b–e	3.67 g–j	7.91 c–f	6.79 ab	2.52 ab
	Areti	31.33 bc	4.00 g–j	7.92 c–f	3.82 e–i	1.35 d–g
	ATS-048/06	27.33 b–g	3.67 g–j	7.43 c–g	5.47 b–e	1.49 c–g
	de Ramellet	28.33 b–f	6.63 c–g	4.26 fgh	7.23 a	2.74 a
	Moneymaker	39.67 a	4.40 g–j	9.23 cde	4.22 d–i	1.35 d–g
	Cherry-INRAE (2)	28 b–g	2.73 g–j	10.39 cd	4.10 d–i	2.52 ab
	Seccagno PSC1-1	34.33 ab	2.80 g–j	14.99 b	4.19 d–i	1.17 efg
	tomataki	28 b–g	3.50 g–j	9.20 cde	4.69 c–i	1.13 efg
	CC_1791 Allungato a Fiasco	22 d–j	2.27 g–j	9.67 cde	4.16 d–i	1.27 d–g
	CC_1665 Pollena	40.67 a	1.83 hij	23.38 a	3.66 f–i	1.21 d–g
	GR-451/04	23.5 c–i	4.15 g–j	6.05 d–h	5.36 b–f	1.81 cde
	Corbarino	23 c–i	2.40 g–j	9.86 cde	6.01 abc	1.81 cde
	Cherry-INRAE (1)	17.67 h–k	1.17 j	15.36 b	3.81 e–i	1.38 d–g
	Cherry-INRAE (3)	23.67 c–i	1.40 ij	17.18 b	6.13 abc	1.37 d–g
	Cherry-INRAE (4)	23 c–i	2.37 g–j	10.87 c	4.43 c–i	1.61 c–g
30 mM NaCl	Chondrokatsari	22.67 d–i	13.00 ab	1.84 h	3.80 e–i	1.26 d–g
	Valldemossa (de)	23.67 c–i	15.50 a	1.63 d	5.01 c–h	1.90 cd
	Areti	22.33 d–i	13.00 ab	1.75 d	3.23 hi	0.91 g
	ATS-048/06	19.67 g–j	10.50 bc	1.87 h	4.44 c–i	1.12 efg
	de Ramellet	17.67 h–k	6.63 c–g	2.04 h	3.55 ghi	1.64 c–f
	Moneymaker	22.67 d–i	11.83 ab	7.96 h	3.74 e–i	0.96 fg
	Cherry-INRAE (2)	23.5 c–i	9.25 b–e	2.71 h	3.05 i	1.90 bcd
	Seccagno PSC1-1	29 b–e	9.00 b–f	3.38 fgh	3.61 f–i	0.97 fg
	tomataki	23 c–i	11.25 ab	2.05 h	4.01 d–i	0.92 g
	CC_1791 Allungato a Fiasco	20.67 e–j	12.17 ab	1.93 h	5.07 c–g	1.23 d–g
	CC_1665 Pollena	23.33 c–i	6.17 d–h	3.79 fgh	4.16 d–i	1.30 d–g
	GR-451/04	11 k	8.97 b–f	1.35 h	5.12 b–g	1.48 c–g
	Corbarino	25 c–h	12.75 ab	2.04 h	4.97 c–h	2.12 bc
	Cherry-INRAE (1)	14 jk	4.83 f–j	2.96 gh	3.51 ghi	1.07 fg
	Cherry-INRAE (3)	20.33 f–j	4.83 f–j	4.25 fgh	4.54 c–i	1.05 fg
	Cherry-INRAE (4)	16 ijk	4.33 g–j	3.77 fgh	3.34 ghi	1.32 d–g
Statistical significance						
Salinity Stress		***	***	***	***	***
Variety		***	***	***	***	***
Salinity Stress × Variety		*	*	***	*	**

Mean values (*n* = 3) followed by different letters within the same column indicate significant differences according to Duncan’s multiple range test (*p* < 0.05). ***, **, and * are significant at *p* < 0.001, *p* < 0.01, and *p* < 0.05, respectively; (Table S1).

**Table 3:** Impact of cultivation under saline conditions on the concentration of micronutrients (Fe, Cu, Mn, and Zn) in the roots of different tomato landraces. In the table, 0.5 mM NaCl refers to control (without NaCl addition), and “30 Mm NaCl” denotes salinity stress.

Roots					
Salinity Stress	Variety	Fe (μg/g)	Cu (μg/g)	Mn (μg/g)	Zn (μg/g)
Interaction					
0.5 mM NaCl	Chondrokatsari	281.61 b–h	14.83 e–h	98.11 a	95.14
	Valldemossa (de)	297.06 b–f	15.55 def	83.41 ghi	77.88
	Areti	269.45 c–h	13.57 e–i	53.73 e–h	75.26
	ATS-048/06	353.86 abc	19.09 bcd	84.65 abc	91.17
	de Ramellet	181.99 f–i	10.65 i–l	42.04 hi	28.61
	Moneymaker	396.85 ab	21.83 ab	71.89 cde	105.62
	Cherry-INRAE (2)	329.33 bcd	15.73 def	44.86 ghi	101.29
	Seccagno PSC1-1	285.8 b–g	15.64 def	45.8 ghi	69.33
	tomataki	462.37 a	23.18 a	97.07 ab	104.97
	CC_1791 Allungato a Fiasco	322.79 b–e	12.4 f–k	64.44 d–g	60.77
	CC_1665 Pollena	293.45 b–g	19.77 abc	97.57 ab	84.68
	GR-451/04	193.97 f–i	10.81 i–l	44.21 ghi	37.98
	Corbarino	236.53 c–i	12.49 f–k	67.47 c–f	61.71
	Cherry-INRAE (1)	263.77 c–h	10.14 i–m	42.65 hi	134.86
	Cherry-INRAE (3)	203.36 e–i	9.79 i–m	54.55 e–h	85.94
	Cherry-INRAE (4)	178.87 f–i	10.19 i–m	37.29 hi	54.60
30 mM NaCl	Chondrokatsari	190.04 f–i	13.07 f–j	57.22 e–h	107.91
	Valldemossa (de)	264.76 c–h	10.88 h–l	48.61 f–i	58.39
	Areti	212.65 d–i	11.99 f–k	37.13 hi	72.72
	ATS-048/06	252.73 c–i	12.91 f–j	56.19 e–h	71.70
	de Ramellet	134.11 i	6.67 m	40.82 hi	39.87
	Moneymaker	128.43 i	15.55 def	49.71 f–i	84.65
	Cherry-INRAE (2)	167.59 ghi	12.25 f–k	32.03 i	89.79
	Seccagno PSC1-1	219.06 d–i	13.43 e–i	50.47 f–i	74.93
	tomataki	250.55 c–i	17.34 cde	78.5 bcd	96.66
	CC_1791 Allungato a Fiasco	177.11 f–i	11.42 g–k	55.57 e–h	71.01
	CC_1665 Pollena	189.47 f–i	12.63 f–k	76.65 cd	69.68
	GR-451/04	171.3 fi	7.17 lm	36.49 hi	38.36
	Corbarino	185.89 f–i	15.38 d–g	51.65 e–i	50.15
	Cherry-INRAE (1)	298.46 b–f	10.33 i–m	44.31 ghi	109.98
	Cherry-INRAE (3)	157.3 hi	9.35 j–m	50.73 f–i	62.33
	Cherry-INRAE (4)	173.76 f–i	8.63 klm	43.64 ghi	55.19
Statistical significance					
Salinity Stress		***	***	***	**
Variety		***	***	***	***
Salinity Stress × Variety		**	**	**	NS

Mean values (*n* = 3) followed by different letters within the same column indicate significant differences according to Duncan’s multiple range test (*p* < 0.05). *** and ** are significant at *p* < 0.001 and *p* < 0.01, respectively; NS = not significant (Table S1).

**Table 4:** Impact of cultivation under saline conditions on the concentration of macronutrients (K, Na, Ca, Mg, and K/Na) in the leaves of different tomato landraces. In the table, 0.5 mM NaCl refers to control (without NaCl addition), and “30 Mm NaCl” denotes salinity stress.

Leaves						
Salinity Stress	Variety	K (mg/g)	Na (mg/g)	K/Na	Ca (mg/g)	Mg (mg/g)
Interaction						
0.5 mM NaCl	Chondrokatsari	47.33	1.79 g–j	27.04 b–f	36.82 a–h	7.17
	Valldemossa (de)	32.67	1.2 hij	29.33 bcd	46.76 a–i	11.17
	Areti	46.67	1.54 g–j	30.48 bcd	52.82 i–k	6.59
	ATS-048/06	22.00	3.67 fg	6.17 efg	57.36 abc	8.33
	de Ramellet	34.00	1.23 hij	27.74 b–e	49.12 b–j	9.45
	Moneymaker	42.33	1.81 g–j	23.38 b–g	47.74 a–d	7.14
	Cherry-INRAE (2)	45.00	1.31 hij	35.15 b	43.07 a	8.03
	Seccagno PSC1-1	43.33	0.83 ij	58.76 a	57.34 c–j	7.77
	tomataki	27.00	1.55 g–j	17.48 b–g	51.72 a–d	4.43
	CC_1791 Allungato a Fiasco	37.00	1.11 ij	33.85 bc	36.64 a–d	8.36
	CC_1665 Pollena	37.33	0.52 j	74.85 a	33.11 jkl	6.44
	GR-451/04	51.00	0.8 ij	71.11 a	58.73 i–k	10.04
	Corbarino	48.33	1.03 ij	56.28 a	37.56 l	11.60
	Cherry-INRAE (1)	41.67	0.76 ij	56.57 a	60.51 a–g	8.24
	Cherry-INRAE (3)	41.67	0.62 j	70.40 a	38.68 e–k	8.21
	Cherry-INRAE (4)	46.67	0.71 ij	67.70 a	37.10 i–k	10.95
30 mM NaCl	Chondrokatsari	28.67	15.33 a	2.01 g	40.61 b–j	6.29
	Valldemossa (de)	18.33	5.67 ef	3.42 g	45.81 c–j	10.01
	Areti	30.00	8.5 cd	3.54 g	54.41 g–k	5.11
	ATS-048/06	18.33	9.5 bc	1.96 g	42.66 b–j	5.35
	de Ramellet	36.33	7.17 de	5.16 fg	46.92 jkl	10.00
	Moneymaker	33.33	10.83 b	3.10 g	36.01 i–k	8.13
	Cherry-INRAE (2)	37.67	6.83 de	5.97 efg	42.48 ab	8.63
	Seccagno PSC1-1	46.67	3.83 fg	12.37 c–g	55.14 d–k	6.25
	tomataki	23.67	10.17 bc	2.37 g	47.87 f–k	3.94
	CC_1791 Allungato a Fiasco	31.33	6.67 de	4.75 g	40.31 a–e	9.15
	CC_1665 Pollena	30.00	2.83 g–j	10.62 d–g	34.28 h–k	7.44
	GR-451/04	40.00	3.07 ghi	13.06 c–g	48.17 g–k	9.60
	Corbarino	42.00	6.83 de	6.34 efg	41.36 kl	9.57
	Cherry-INRAE (1)	32.33	3.5 gh	9.36 d–g	59.62 a–f	8.72
	Cherry-INRAE (3)	35.67	2.53 g–j	14.08 b–g	32.80 f–k	7.66
	Cherry-INRAE (4)	44.00	2.67 g–j	16.60 b–g	0.16 l	10.16
Statistical significance						
Salinity Stress		***	***	***	**	NS
Variety		***	***	***	***	***
Salinity Stress × Variety		NS	***	***	*	NS

Mean values (*n* = 3) followed by different letters within the same column indicate significant differences according to Duncan’s multiple range test (*p*< 0.05). ***, **, and * are significant at *p* < 0.001, *p* < 0.01, and *p* < 0.05, respectively; NS = not significant (Table S1).

**Table 5:** Impact of cultivation under saline conditions on the concentration of micronutrients (Fe, Cu, Mn, and Zn) in the leaves of different tomato landraces. In the table, 0.5 mM NaCl refers to control (without NaCl addition), and “30 Mm NaCl” denotes salinity stress.

Leaves					
Salinity Stress	Variety	Fe (μg/g)	Cu (μg/g)	Mn (μg/g)	Zn (μg/g)
Interaction					
0.5 mM NaCl	Chondrokatsari	68.64 f–k	11.58 e–j	239.11 c–h	53.33 cde
	Valldemossa (de)	79.57 c–g	13.1 e–h	240.41 c–h	47.27 c–h
	Areti	64.69 h–l	13.8 d–g	231.62 c–j	54.52 cd
	ATS-048/06	62.34 jkl	8.04 hij	234.35 c–i	52.93 cde
	de Ramellet	64.93 h–l	6.79 j	200.1 e–l	35.87 e–j
	Moneymaker	53.27 lmn	8.53 g–j	268.95 a–e	34.69 f–k
	Cherry-INRAE (2)	72.34 e–j	18.96 c	335.07 a	53.73 cde
	Seccagno PSC1-1	78.66 c–h	10.24 e–j	262.38 b–f	42.2 d–i
	tomataki	70.37 f–j	10.46 e–j	273.66 a–e	41.26 d–i
	CC_1791 Allungato a Fiasco	64.36 h–l	9.59 f–j	280.99 a–d	54.41 cd
	CC_1665 Pollena	92.3 abc	13.57 d–g	174.58 g–m	29.73 h–k
	GR-451/04	54.87 k–n	7.52 ij	156.73 j–m	51.42 c–f
	Corbarino	65.51 g–l	10.29 e–j	148.57 k–n	41.36 d–i
	Cherry-INRAE (1)	64.04 h–l	46.1 a	243.95 c–h	53.16 cde
	Cherry-INRAE (3)	73.15 e–j	13.93 d–g	167.57 h–m	34.69 f–k
	Cherry-INRAE (4)	74.31 e–j	11.05 e–j	158.45 i–m	44.81 d–h
30 mM NaCl	Chondrokatsari	44.66 n	8.58 g–j	160.76 i–m	49.3 c–g
	Valldemossa (de)	91.83 a–d	12.53 e–i	233.71 c–i	26.39 ijk
	Areti	83.13 a–f	34.07 b	184.46 g–m	38.47 d–i
	ATS-048/06	53.4 lmn	11.45 e–j	190.97 f–l	40.32 d–i
	de Ramellet	77.8 d–i	7.28 ij	80.99 n	18.13 k
	Moneymaker	47.66 mn	9.63 f–j	169.58 h–m	45.65 c–h
	Cherry-INRAE (2)	61.05 j–m	18.32 cd	248.02 b–g	63.4 bc
	Seccagno PSC1-1	79.69 c–g	14.83 c–f	316.73 ab	112.54 a
	tomataki	55.42 k–n	8.14 hij	148.54 k–n	39.06 d–i
	CC_1791 Allungato a Fiasco	66.29 g–l	9.89 f–j	293.21 abc	54.78 cd
	CC_1665 Pollena	94.12 ab	11.87 e–j	160.33 i–m	35.54 e–k
	GR-451/04	82.24 b–f	7.86 hij	128.75 lmn	18.99 jk
	Corbarino	86.39 a–e	9.97 f–j	111.72 mn	32.89 g–k
	Cherry-INRAE (1)	63.45 i–l	15.46 cde	213.98 d–k	55.03 cd
	Cherry-INRAE (3)	96.61 a	13.9 d–g	184.31 g–m	32.79 g–k
	Cherry-INRAE (4)	77.93 d–i	13.18 d–h	174.09 g–m	73.12 b
Statistical significance					
Salinity Stress		*	NS	***	NS
Variety		***	***	***	***
Salinity Stress × Variety		***	***	**	***

Mean values (*n* = 3) followed by different letters within the same column indicate significant differences according to Duncan’s multiple range test (*p* < 0.05). ***, **, and * are significant at *p* < 0.001, *p* < 0.01, and *p* < 0.05, respectively; NS = not significant (Table S1).

**Table 6:** Impact of cultivation under saline conditions on the concentration of macronutrients (K, Na, Ca, Mg, and K/Na) in the fruit of different tomato landraces. In the table, 0.5 mM NaCl refers to control (without NaCl addition), and “30 Mm NaCl” denotes salinity stress.

Fruit						
Salinity Stress	Variety	K (mg/g)	Na (mg/g)	K/Na	Ca (mg/g)	Mg (mg/g)
Interaction						
0.5 mM NaCl	Chondrokatsari	37.5 a–f	0.54 j–m	73.57	0.18 cd	1.63 cde
	Valldemossa (de)	37 c–f	0.68 h–l	57.72	0.01 g	1.52 efg
	Areti	33 f–k	0.38 m	89.02	0.29 a	1.30 hi
	ATS-048/06	41.75 ab	0.65 i–m	66.02	0.28 a	1.79 abc
	de Ramellet	38.25 a–d	0.75 hij	51.50	0.01 g	1.80 abc
	Moneymaker	36 d–g	0.71 h–k	51.92	0.01 g	1.53 efg
	Cherry-INRAE (2)	36 d–g	0.58 i–m	65.21	0.25 ab	1.53 efg
	Seccagno PSC1-1	35.8 d–g	0.62 i–m	57.92	0.16 cde	1.35 ghi
	tomataki	41.75 ab	0.75 g–j	55.67	0.14 def	1.88 ab
	CC_1791 Allungato a Fiasco	32.25 g–k	0.42 klm	76.79	0.12 ef	1.35 ghi
	CC_1665 Pollena	26 mno	0.42 klm	68.52	0.20 bc	1.33 ghi
	GR-451/04	28.75 k–n	0.38 m	79.63	0.26 a	1.40 f–i
	Corbarino	30.25 i–m	0.38 lm	84.31	0.17 cde	1.45 e–i
	Cherry-INRAE (1)	26.75 l–o	0.36 m	76.97	0.14 def	1.31 hi
	Cherry-INRAE (3)	25.75 no	0.56 j–m	59.41	0.20 bc	1.60 def
	Cherry-INRAE (4)	23.33 op	0.37 m	64.73	0.19 cd	1.50 e–h
30 mM NaCl	Chondrokatsari	34.25 d–i	1.64 b	21.22	0.21 bc	1.52 efg
	Valldemossa (de)	34 d–i	1.29 cde	27.13	0.01 g	1.25 ij
	Areti	33.25 e–j	1.13 def	30.35	0.18 cd	1.42 e–i
	ATS-048/06	41 abc	1.55 bc	26.88	0.17 cde	1.96 a
	de Ramellet	32.25 g–k	1.35 cd	24.40	0.01 g	1.45 e–i
	Moneymaker	33.75 d–i	1.40 bcd	24.72	0.01 g	1.50 e–h
	Cherry-INRAE (2)	29 j–n	2.09 a	13.87	0.24 ab	1.85 ab
	Seccagno PSC1-1	37.75 a–e	1.03 efg	37.28	0.20 bc	1.44 e–i
	tomataki	35 d–h	1.97 a	17.83	0.14 def	1.76 bcd
	CC_1791 Allungato a Fiasco	34 d–i	0.87 f–i	40.41	0.11 f	1.46 e–i
	CC_1665 Pollena	21 p	0.41 klm	52.03	0.17 cde	1.09 j
	GR-451/04	30.75 h–l	1.13 def	32.81	0.13 def	1.33 ghi
	Corbarino	29.25 j–n	0.65 h–m	48.04	0.13 def	1.08 j
	Cherry-INRAE (1)	25.75 no	0.49 j–m	52.80	0.12 ef	1.08 j
	Cherry-INRAE (3)	29 j–n	0.94 fgh	31.18	0.20 bc	1.57 ef
	Cherry-INRAE (4)	22.67 op	0.61 i–m	37.65	0.16 c–f	1.41 f–i
Statistical significance						
Salinity Stress		***	***	***	***	**
Variety		***	***	***	***	***
Salinity Stress × Variety		***	***	NS	***	***

Mean values (*n* = 3) followed by different letters within the same column indicate significant differences according to Duncan’s multiple range test (*p* < 0.05). *** and ** are significant at *p* < 0.001 and *p* < 0.01, respectively; NS = not significant (Table S1).

**Table 7:** Impact of cultivation under saline conditions on the concentration of micronutrients (Fe, Cu, Mn, and Zn) in the fruit of different tomato landraces. In the table, 0.5 mM NaCl refers to control (without NaCl addition), and “30 Mm NaCl” denotes salinity stress.

Fruit					
Salinity Stress	Variety	Fe (μg/g)	Cu (μg/g)	Mn (μg/g)	Zn (μg/g)
Interaction					
0.5 mM NaCl	Chondrokatsari	37.55 h–m	7.48 f–j	12.53 d–g	20.9 g–m
	Valldemossa (de)	47.78 c–i	3.73 m	15.21 c	20.16 j–m
	Areti	43.78 e–k	7.52 f–j	11.66 f–i	20.38 i–m
	ATS-048/06	31.45 lm	6.13 jkl	13.69 cde	21.15 f–m
	de Ramellet	71.45 a	7.08 h–k	13.34 def	36.56 a
	Moneymaker	56.39 cd	6.6 i–l	14.06 cd	25.13 def
	Cherry-INRAE (2)	46.55 c–j	8.51 e–h	19.71 a	24.39 d–i
	Seccagno PSC1-1	68.50 a	7.14 h–k	12.82 def	32.98 b
	tomataki	34.47 klm	7.85 e–i	11.89 fgh	23.82 e–k
	CC_1791 Allungato a Fiasco	44.64 d–k	14.05 ab	8.89 k–p	20.49 h–m
	CC_1665 Pollena	54.19 cde	12.91 bc	8.03 m–q	21.23 f–m
	GR-451/04	46.55 c–j	3.78 m	8.43 l–p	24.63 d–g
	Corbarino	49.02 c–h	14.32 a	7.49 opq	24.17 e–j
	Cherry-INRAE (1)	46.30 c–k	8.39 e–h	7.57 opq	19.87 klm
	Cherry-INRAE (3)	55.00 cde	8.70 efg	10.28 h–k	30.04 bc
	Cherry-INRAE (4)	50.47 c–g	8.6 e–h	8.61 k–p	24.49 d–h
30 mM NaCl	Chondrokatsari	39.95 g–l	6.31 jkl	9.60 j–n	19.18 lmn
	Valldemossa (de)	35.76 i–m	3.54 m	11.98 e–h	18.40 mn
	Areti	27.25 m	7.12 h–k	9.82 j–m	17.77 mn
	ATS-048/06	35.2 j–m	7.62 f–j	12.57 d–g	22.45 e–l
	de Ramellet	45.07 d–k	3.57 m	9.30 j–o	31.25 bc
	Moneymaker	41.92 f–l	5.87 kl	12.32 d–g	22.93 e–l
	Cherry-INRAE (2)	40.27 g–l	8.36 e–h	17.71 b	23.72 e–k
	Seccagno PSC1-1	67.45 ab	7.21 g–k	13.71 cde	32.47 b
	tomataki	31.88 lm	7.55 f–j	10.94 g–j	22.82 e–l
	CC_1791 Allungato a Fiasco	47.11 c–j	14.29 a	9.14 k–p	22.48 e–l
	CC_1665 Pollena	44.02 e–k	11.56 d	5.78 rs	15.53 n
	GR-451/04	26.39 m	5.31 l	6.28 qrs	20.18 j–m
	Corbarino	48.77 c–h	12.03 cd	5.35 s	21.64 f–m
	Cherry-INRAE (1)	44.7 d–k	8.93 e–f	7.37 pqr	21.13 f–m
	Cherry-INRAE (3)	58.02 bc	7.38 g–j	10.10 i–l	28.09 cd
	Cherry-INRAE (4)	52.53 c–f	9.12 e	7.88 n–q	25.81 de
Statistical significance					
Salinity Stress		***	**	***	***
Variety		***	***	***	***
Salinity Stress × Variety		***	***	***	*

Mean values (*n* = 3) followed by different letters within the same column indicate significant differences according to Duncan’s multiple range test (*p* < 0.05). ***, **, and * are significant at *p* < 0.001, *p* < 0.01, and *p* < 0.05, respectively (Table S1).

**Table 8:** Seed source and name of the cultivated tomato landraces.

Variety	Provider
Moneymaker (Reference)	INRA ^1^
de Ramellet	UIB ^2^
Valldemossa (de)	UIB ^2^
Seccagno PSC1-1	UIB/UNITO ^3^
CC_1791 Allungato a Fiasco	UIB/UNITO ^3^
CC_1665 Pollena	UIB/UNITO ^3^
Corbarino	UIB/UNITO ^3^
GR-451/04	AUA ^4^
ATS-048/06	AUA ^4^
tomataki	AUA ^4^
Chondrokatsari	AUA ^4^
Areti	AUA ^4^
Cherry-INRAE (1)	INRAE ^5^
Cherry-INRAE (2)	INRAE ^5^
Cherry-INRAE (3)	INRAE ^5^
Cherry-INRAE (4)	INRAE ^5^

^1^ Institut National de la Recherche Agronomique. ^2^ University of the Balearic Islands. ^3^ University of the Balearic Islands/University of Turin. ^4^ Agricultural University of Athens. ^5^ National Research Institute for Agriculture, Food, and the Environment.

**Table 9:** Nutrient concentrations in the nutrient solution supplied to the plants during the vegetative and reproductive growth phases.

Nutrient	Drainage Solution (3 October 2021)	Vegetative Phase (3 November 2021)	Reproductive Phase (4 May 2021)	Reproductive Phase (29 April 2021)	Reproductive Phase (5 October 2021)	Unit
NO_3_^−^	16.76	14.70	13.80	14.09	13.39	mM
K^+^	7.50	8.88	8.92	9.01	7.81	mM
Ca^2+^	9.40	5.36	5.83	5.72	4.93	mM
Mg^2+^	4.25	2.51	2.47	2.60	2.14	mM
SO_4_^2−^	7.19	3.59	4.24	3.95	2.44	mM
H_2_PO_4_^−^	1.20	1.42	1.40	1.60	1.60	mM
NH_4_^+^	1.34	1.28	1.36	1.23	1.21	mM
Fe	15.00	20.00	18.50	18.50	17.21	μM
Mn^++^	10.00	10.00	9.50	9.50	8.84	μM
Zn^++^	5.00	6.50	6.30	8.00	8.00	μM
B	30.00	35.00	35.60	35.60	33.11	μM
Cu^++^	0.75	0.80	0.80	0.80	0.74	μM
Mo	0.50	0.50	0.50	0.50	0.47	μΜ
Cl^−^	4.00	2.80	3.40	3.40	3.40	μΜ

## Data Availability

Not applicable.

## Supplementary Materials

The following supporting information can be downloaded at: https://www.mdpi.com/article/10.3390/plants12203551/s1, Table S1: Standard errors of means (*n* = 3) of the concentration of macro- (K, Na, Ca, Mg and K/Na) and micro-nutrients (Fe, Cu, Mn, Zn) in roots of different tomato landraces.

## References

[1] Sonneveld C. (2000). Effects of Salinity on Substrate Grown Vegetables and Ornamentals in Greenhouse Horticulture.

[2] Hopmans J.W., Qureshi A.S., Kisekka I., Munns R., Grattan S.R., Rengasamy P., Ben-Gal A., Assouline S., Javaux M., Minhas P.S. (2021). Critical Knowledge Gaps and Research Priorities in Global Soil Salinity. Adv. Agron..

[3] Khalid M.F., Huda S., Yong M., Li L., Li L., Chen Z.H., Ahmed T. (2022). Alleviation of Drought and Salt Stress in Vegetables: Crop Responses and Mitigation Strategies. Plant Growth Regul..

[4] Munns R. (2005). Genes and Salt Tolerance: Bringing Them Together. New Phytol..

[5] Savvas D., Gianquinto G., Tuzel Y., Gruda N. (2013). Soilless Culture.

[6] Kumar S., Li G., Yang J., Huang X., Ji Q., Liu Z., Ke W., Hou H. (2021). Effect of Salt Stress on Growth, Physiological Parameters, and Ionic Concentration of Water Dropwort (*Oenanthe javanica*) Cultivars. Front. Plant Sci..

[7] Magán J.J., Gallardo M., Thompson R.B., Lorenzo P. (2008). Effects of Salinity on Fruit Yield and Quality of Tomato Grown in Soil-Less Culture in Greenhouses in Mediterranean Climatic Conditions. Agric. Water Manag..

[8] Cuartero J., Fernández-Muñoz R. (1998). Tomato and Salinity. Sci. Hortic..

[9] Gama P.B.S., Inanaga S., Tanaka K., Nakazawa R. (2007). Physiological Response of Common Bean (*Phaseolus vulgaris* L.) Seedlings to Salinity Stress. Afr. J. Biotechnol..

[10] Keutgen A.J., Pawelzik E. (2009). Impacts of NaCl Stress on Plant Growth and Mineral Nutrient Assimilation in Two Cultivars of Strawberry. Environ. Exp. Bot..

[11] FAO (2022). The State of Food and Agriculture 2022. Leveraging Automation in Agriculture for Transforming Agrifood Systems.

[12] Tognoni F., Serra G. (2003). Trends in Process Technologies and Products. Acta Hortic..

[13] Maas E.V., Poss J.A., Hoffman G.J. (1986). Salinity Sensitivity of Sorghum at Three Growth Stages. Irrig. Sci..

[14] Singh J., Sastry E.V.D., Singh V. (2012). Effect of Salinity on Tomato (*Lycopersicon esculentum* Mill.) during Seed Germination Stage. Physiol. Mol. Biol. Plants.

[15] Ladewig P., Trejo-Téllez L.I., Servín-Juárez R., Contreras-Oliva A., Gómez-Merino F.C. (2021). Growth, Yield and Fruit Quality of Mexican Tomato Landraces in Response to Salt Stress. Not. Bot. Horti Agrobot. Cluj-Napoca.

[16] Maas E.V., Hoffman G.J. (1977). Crop Salt Tolerance—Current Assessment. ASCE J. Irrig. Drain. Div..

[17] Brasiliano Campos C.A., Fernandes P.D., Gheyi H.R., Blanco F.F., Gonçalves C.B., Ferreira Campos S.A. (2006). Yield and Fruit Quality of Industrial Tomato under Saline Irrigation. Sci. Agric..

[18] Villa T.C.C., Maxted N., Scholten M., Ford-Lloyd B. (2005). Defining and Identifying Crop Landraces. Plant Genet. Resour..

[19] Almekinders C.J., Louwaars N.P. (1999). Farmers’ Seed Production: New Approaches and Practices. Intermed. Technol..

[20] Frankel O.H., Brown A.H., Burdon J.J. (1995). The Conservation of Plant Biodiversity.

[21] Galmés J., Ochogavía J.M., Gago J., Roldán E.J., Cifre J., Conesa M.À. (2013). Leaf Responses to Drought Stress in Mediterranean Accessions of Solanum Lycopersicum: Anatomical Adaptations in Relation to Gas Exchange Parameters. Plant Cell Environ..

[22] Assimakopoulou A., Nifakos K., Salmas I., Kalogeropoulos P. (2015). Growth, Ion Uptake, and Yield Responses of Three Indigenous Small-Sized Greek Tomato (Lycopersicon esculentum L.) Cultivars and Four Hybrids of Cherry Tomato under NaCl Salinity Stress. Commun. Soil Sci. Plant Anal..

[23] Raza M.A., Saeed A., Munir H., Ziaf K., Shakeel A., Saeed N., Munawar A., Rehman F. (2017). Screening of Tomato Genotypes for Salinity Tolerance Based on Early Growth Attributes and Leaf Inorganic Osmolytes. Arch. Agron. Soil Sci..

[24] Hasegawa P.M., Bressan R.A. (2000). Plant Cellular and Molecular Responses to High Salinity. Annu. Rev. Plant Physiol. Plant Mol. Biol..

[25] Bolarín M.C., Cano E.A., Estañ M.T., Caro M. (1993). Growth, Fruit Yield, and Ion Concentration in Tomato Genotypes after Pre- and Post-Emergence Salt Treatments. J. Am. Soc. Hortic. Sci..

[26] Romero-Aranda R., Soria T., Cuartero J. (2001). Tomato Plant-Water Uptake and Plant-Water Relationships under Saline Growth Conditions. Plant Sci..

[27] Isayenkov S.V., Maathuis F.J.M. (2019). Plant Salinity Stress: Many Unanswered Questions Remain. Front. Plant Sci..

[28] McCall D., Brazaitytė A. (1997). Salinity Effects on Seedling Growth and Floral Initiation in the Tomato. Acta Agric. Scand. Sect. B Soil Plant Sci..

[29] Adams P. (1990). Effects of Watering on the Yield, Quality and Composition of Tomatoes Grown in Bags of Peat. J. Hortic. Sci..

[30] Azarmi R., Taleshmikail R.D., Gikloo A. (2010). Effects of Salinity on Morphological and Physiological Changes and Yield of Tomato in Hydroponics System. J. Food Agric. Environ..

[31] Hajiaghaei-Kamrani M., Khoshvaghti H., Hosseinniya H. (2013). Effects of Salinity and Hydroponic Growth Media on Growth Parameters in Tomato (*Lycopersicon esculentum* Mill.). Int. J. Agron. Plant Prod..

[32] Cruz V., Cuartero J. Effects of Salinity at Several Developmental Stages of Six Genotypes of Tomato (*Lycopersicon* spp.). Proceedings of the XIth Eucarpia Meeting on Tomato Genetics and Breeding.

[33] Bolarín M.C., Fernández F.G., Cruz V., Cuartero J. (1991). Salinity Tolerance in Four Wild Tomato Species Using Vegetative Yield-Salinity Response Curves. J. Am. Soc. Hortic. Sci..

[34] Saranga Y., Zamir D., Marani A., Rudich J. (1991). Breeding Tomatoes for Salt Tolerance: Field Evaluation of Lycopersicon Germplasm for Yield and Dry-Matter Production. J. Am. Soc. Hortic. Sci..

[35] Rodríguez-Ortega W.M., Martínez V., Nieves M., Simón I., Lidón V., Fernandez-Zapata J.C., Martinez-Nicolas J.J., Cámara-Zapata J.M., García-Sánchez F. (2019). Agricultural and Physiological Responses of Tomato Plants Grown in Different Soilless Culture Systems with Saline Water under Greenhouse Conditions. Sci. Rep..

[36] Caro M., Cruz V., Cuartero J., Estañ M.T., Bolarin M.C. (1991). Salinity Tolerance of Normal-Fruited and Cherry Tomato Cultivars. Plant Soil.

[37] Liu F.Y., Li K.T., Yang W.J. (2014). Differential Responses to Short-Term Salinity Stress of Heat-Tolerant Cherry Tomato Cultivars Grown at High Temperatures. Hortic. Environ. Biotechnol..

[38] AA H. (2017). Effect of Irrigation with Different Levels of Saline Water Type on Husk Tomato Productivity. Adv. Plants Agric. Res..

[39] Psarras G., Bertaki M., Chartzoulakis K. (2008). Response of Greenhouse Tomato to Salt Stress and K+ Supplement. Plant Biosyst..

[40] Shiyab S.M., Shatnawi M.A., Shibli R.A., Al Smeirat N.G., Ayad J., Akash M.W. (2013). Growth, Nutrient Acquisition, and Physiological Responses of Hydroponic Grown Tomato to Sodium Chloride Salt Induced Stress. J. Plant Nutr..

[41] Babu M.A., Singh D., Gothandam K.M. (2012). The Effect of Salinity on Growth, Hormones and Mineral Elements in Leaf and Fruit of Tomato Cultivar PKM1. J. Anim. Plant Sci..

[42] Alfocea F.P., Estañ M.T., Caro M., Bolarín M.C. (1993). Response of Tomato Cultivars to Salinity. Plant Soil.

[43] Kusvuran S., Yasar F., Ellialtioglu S., Abak K. (2007). Utilizing Some of Screening Methots in Order to Determine of Tolerance of Salt Stress in the Melon (*Cucumis melo* L.). Res. J. Agric. Biol. Sci..

[44] Munns R. (2002). Comparative Physiology of Salt and Water Stress. Plant Cell Environ..

[45] San-Martín-Hernández C., Gómez-Merino F.C., Rivera-Vargas G., Saucedo-Veloz C., Vaquera-Huerta H., Trejo-Téllez L.I. (2022). Tomato Fruit Quality between Clusters Is Differentially Affected By Nitrogen and Potassium Supply. Rev. Fitotec. Mex..

[46] Marschner H. (1995). Mineral Nutrition of Higher Plants.

[47] Li Y. (2009). Physiological Responses of Tomato Seedlings (*Lycopersicon esculentum*) to Salt Stress. Modern Appl. Sci..

[48] Adams P., Ho L.C. (1995). Uptake and Distribution Ofnutrients in Relation to Tomato Fruit Quality. Acta Hortic..

[49] Taffouo V.D., Nouck A.H., Dibong S.D., Amougou A. (2010). Effects of Salinity Stress on Seedlings Growth, Mineral Nutrients and Total Chlorophyll of Some Tomato (*Lycopersicum esculentum* L.) Cultivars. Afr. J. Biotechnol..

[50] Mansour M.M.F., Salama K.H.A., Al-Mutawa M.M. (2003). Transport Proteins and Salt Tolerance in Plants. Plant Sci..

[51] Zeng L., Poss J.A., Wilson C., Draz A.S.E., Gregorio G.B., Grieve C.M. (2003). Evaluation of Salt Tolerance in Rice Genotypes by Physiological Characters. Euphytica.

[52] Shabala S., Cuin T.A. (2008). Potassium Transport and Plant Salt Tolerance. Physiol. Plant..

[53] Siddiky M.A., Khan M.S., Rahman M.M., Uddin M.K. (2015). Performance of Tomato (*Lycopersicon esculentum* Mill.) Germplasm to Salinity Stress. Bangladesh J. Bot..

[54] Tuna A.L., Kaya C., Ashraf M., Altunlu H., Yokas I., Yagmur B. (2007). The Effects of Calcium Sulphate on Growth, Membrane Stability and Nutrient Uptake of Tomato Plants Grown under Salt Stress. Environ. Exp. Bot..

[55] Shabani E., Tabatabaei S.J., Bolandnazar S., Ghasemi K. (2012). Vegetative Growth and Nutrient Uptake of Salinity Stressed Cherry Tomato in Different Calcium and Potassium Level. Int. Res. J. Appl. Basic Sci..

[56] Malone M., Andrews J. (2001). The Distribution of Xylem Hydraulic Resistance in the Fruiting Truss of Tomato. Plant Cell Environ..

[57] Grattan S., Grieve C., Pessarakli M. (1999). Mineral Nutrient Acquisition and Response by Plants Grown in Saline Environments. Handbook of Plant and Crop Stress.

[58] Subbarao G.V., Johansen C., Jana M.K., Kumar Rao J.V.D.K. (1990). Effects of the Sodium/Calcium Ratio in Modifying Salinity Response of Pigeonpea (*Cajanus cajan*). J. Plant Physiol..

[59] Yunus Q., Zari M. (2017). Effect of Exogenous Silicon on Ion Distribution of Tomato Plants Under Salt Stress. Commun. Soil Sci. Plant Anal..

[60] Li H., Zhu Y., Hu Y., Han W., Gong H. (2015). Beneficial Effects of Silicon in Alleviating Salinity Stress of Tomato Seedlings Grown under Sand Culture. Acta Physiol. Plant..

[61] Shibli R.A., Kushad M., Yousef G.G., Lila M.A. (2007). Physiological and Biochemical Responses of Tomato Microshoots to Induced Salinity Stress with Associated Ethylene Accumulation. Plant Growth Regul..

[62] Al-Karaki G.N., Hammad R. (2001). Mycorrhizal Influence on Fruit Yield and Mineral Content of Tomato Grown under Salt Stress. J. Plant Nutr..

[63] Nouck A.E., Taffouo V.D., Tsoata E., Dibong D.S., Nguemezi S.T., Gouado I., Youmbi E. (2016). Growth, Biochemical Constituents, Micronutrient Uptake and Yield Response of Six Tomato (*Lycopersicum esculentum* L.) Cultivars Grown under Salinity Stress. J. Agron..

[64] Fuentes J.E., Castellanos B.F., Martínez E.N., Ortiz W.A., Mendoza A.B., Macías J.M. (2022). Outcomes of Foliar Iodine Application on Growth, Minerals and Antioxidants in Tomato Plants under Salt Stress. Folia Hortic..

[65] Alam P., Arshad M., Al-Kheraif A.A., Azzam M.A., Al Balawi T. (2022). Silicon Nanoparticle-Induced Regulation of Carbohydrate Metabolism, Photosynthesis, and ROS Homeostasis in Solanum Lycopersicum Subjected to Salinity Stress. ACS Omega.

[66] El-Fouly M.M., Moubarak Z.M., Salama Z.A. (2002). Micronutrient Foliar Application Increases Salt Tolerance of Tomato Seedlings. Acta Hortic..

[67] Kowalska I., Smoleń S. (2013). Effect of Foliar Application of Salicylic Acid on the Response of Tomato Plants to Oxidative Stress and Salinity. J. Elem..

[68] Carmassi G., Incrocci L., Maggini R., Malorgio F., Tognoni F., Pardossi A. (2005). Modeling Salinity Build-up in Recirculating Nutrient Solution Culture. J. Plant Nutr..

