# Supplementary material for: Assessment of Growth, Yield, and Nutrient Uptake of Mediterranean Tomato Landraces in Response to Salinity Stress
Source: Plants (Basel). 2023 Oct 12;12(20):3551. doi: 10.3390/plants12203551 (PMC10610299; doi:10.3390/plants12203551)
Supplement: Supplementary file 1 [file plants-12-03551-s001.zip › plants-2632711-supplementary.pdf]

Table S1. Standard errors of means (n =3) of the concentration of macro- (K, Na, Ca, Mg and K/Na) and micro- nutrients (Fe, Cu, Mn, Zn) in roots of different tomato landraces.

Salinity Stress	Variety	K (mg/g)	Na (mg/g)	K/Na	Ca (mg/g)	Mg (mg/g)	Fe (μg/g)	Cu (μg/g)	Mn (μg/g)	Zn (μg/g)
					Roots					
					Interraction					
0.5 mM NaCl	**Chondrokatsari**	2.40	1.12	1.18	0.27	0.30	37.89	1.12	7.24	11.52
	**Valldemossa (de)**	1.53	0.09	0.38	0.45	0.38	35.81	0.40	7.95	8.33
	**Areti**	3.93	0.66	0.29	0.23	0.07	28.56	0.77	3.05	4.54
	**ATS-048/06**	4.33	0.05	1.09	0.59	0.26	57.42	1.66	5.14	4.84
	**de Ramellet**	2.33	0.37	0.19	0.35	0.33	25.16	0.64	9.17	2.78
	**Moneymaker**	2.03	0.61	0.77	0.51	0.19	35.26	0.16	3.17	9.17
	**Cherry-INRAE (2)**	1.15	0.30	0.67	0.39	0.27	41.34	1.32	4.97	5.91
	**Seccagno PSC1-1**	2.73	0.96	4.07	0.17	0.24	42.31	1.80	2.68	10.53
	**tomataki**	0.58	0.91	2.35	0.49	0.16	33.89	1.83	8.24	12.10
	**CC_1791 Allungato a Fiasco**	3.21	0.35	1.04	0.37	0.20	14.78	0.59	7.84	6.82
	**CC_1665 Pollena**	3.53	0.33	4.02	0.34	0.21	40.20	1.29	2.36	6.85
	**GR-451/04**	0.87	0.66	1.22	0.85	0.08	45.60	0.27	7.07	6.38
	**Corbarino**	3.79	0.50	1.39	0.48	0.13	69.47	0.75	6.05	8.46
	**Cherry-INRAE (1)**	1.45	0.10	1.98	0.88	0.25	66.94	1.21	5.25	3.10
	**Cherry-INRAE (3)**	2.91	0.17	2.12	0.12	0.13	40.64	0.92	6.68	6.76

	**Cherry-INRAE (4)**	3.79	0.69	2.11	0.74	0.22	25.99	0.87	3.48	6.26
**30 mM NaCl**	**Chondrokatsari**	2.60	1.76	0.38	0.13	0.17	16.71	1.93	3.45	8.45
	**Valldemossa (de)**	1.33	2.29	0.34	0.77	0.10	58.17	0.65	6.67	1.94
	**Areti**	1.20	1.04	0.21	0.17	0.01	26.09	1.05	1.61	6.00
	**ATS-048/06**	2.33	0.58	0.18	0.18	0.14	24.14	1.42	5.87	1.60
	**de Ramellet**	3.48	2.77	0.64	0.70	0.22	14.83	1.22	5.59	9.85
	**Moneymaker**	2.03	1.88	0.15	0.26	0.12	12.92	2.42	3.10	6.87
	**Cherry-INRAE (2)**	1.44	1.88	0.42	0.17	0.07	26.85	0.62	4.05	13.52
	**Seccagno PSC1-1**	2.52	1.61	0.48	0.11	0.04	32.60	0.87	6.44	3.79
	**tomataki**	0.00	0.43	0.08	0.44	0.07	20.01	0.63	9.23	11.32
	**CC_1791 Allungato a Fiasco**	1.45	3.56	0.39	0.50	0.16	20.59	1.10	1.70	10.59
	**CC_1665 Pollena**	0.88	0.17	0.23	0.71	0.17	52.90	1.39	4.95	9.80
	**GR-451/04**	1.53	2.50	0.41	1.02	0.38	42.11	1.20	3.01	7.85
	**Corbarino**	3.46	2.74	0.18	0.99	0.11	18.66	1.50	10.66	7.93
	**Cherry-INRAE (1)**	3.21	1.17	0.23	0.29	0.18	16.88	1.11	8.30	9.80
	**Cherry-INRAE (3)**	1.76	0.60	0.24	0.38	0.15	21.04	1.06	9.81	9.61
	**Cherry-INRAE (4)**	2.00	0.33	0.65	0.33	0.25	14.76	0.57	5.16	5.04

Table S2. Standard errors of means (n =3) of the concentration of macro- (K, Na, Ca, Mg and K/Na) and micro- nutrients (Fe, Cu, Mn, Zn) in leaves of different tomato landraces.

		Leaves								
**Salinity Stress**	**Variety**	**K (mg/g)**	**Na (mg/g)**	**K/Na**	**Ca (mg/g)**	**Mg (mg/g)**	**Fe (μg/g)**	**Cu (μg/g)**	**Mn (μg/g)**	**Zn (μg/g)**
		**Interraction**								
**0.5 mM NaCl**	**Chondrokatsari**	9.13	0.11	6.54	3.77	1.20	4.16	1.53	10.74	6.17
	**Valldemossa (de)**	6.39	0.32	5.84	2.66	0.76	0.83	0.90	12.15	4.57
	**Areti**	1.86	0.12	1.18	3.81	0.77	2.51	1.01	5.68	4.43
	**ATS-048/06**	1.53	0.33	0.96	1.26	0.74	5.89	0.51	12.39	7.19
	**de Ramellet**	1.00	0.05	0.36	1.53	0.51	0.59	0.77	9.25	3.89
	**Moneymaker**	5.81	0.10	3.20	8.42	0.91	3.96	0.09	16.81	3.21
	**Cherry-INRAE (2)**	5.51	0.19	4.95	2.76	0.37	3.85	1.20	3.25	12.54
	**Seccagno PSC1-1**	3.28	0.18	15.69	3.23	1.13	0.74	0.72	30.03	6.55
	**tomataki**	3.21	0.06	2.12	1.33	0.58	4.63	1.35	13.46	5.60
	**CC_1791 Allungato a Fiasco**	1.00	0.08	3.07	3.54	0.59	0.99	1.44	13.37	5.71
	**CC_1665 Pollena**	2.73	0.08	11.92	1.53	0.04	3.73	0.83	14.44	2.65
	**GR-451/04**	6.08	0.20	17.86	2.98	0.94	3.95	0.47	11.49	3.14
	**Corbarino**	1.33	0.32	14.74	2.09	0.80	2.45	1.33	18.31	2.72
	**Cherry-INRAE (1)**	2.60	0.07	9.23	5.55	1.29	4.27	6.72	33.55	5.51
	**Cherry-INRAE (3)**	4.26	0.10	10.83	1.67	0.61	3.06	0.33	7.07	3.03
	**Cherry-INRAE (4)**	1.67	0.06	9.26	3.63	0.74	3.63	1.02	16.86	5.33

30 mM NaCl	**Chondrokatsari**	6.44	2.17	0.60	2.72	0.24	5.44	1.68	27.68	6.01
	**Valldemossa (de)**	0.88	0.88	0.60	6.09	1.38	3.83	1.25	11.00	1.62
	**Areti**	1.00	0.29	0.23	3.20	0.49	3.60	1.82	38.40	4.55
	**ATS-048/06**	1.45	0.76	0.26	1.89	0.17	2.49	1.10	11.72	5.00
	**de Ramellet**	3.84	1.01	0.52	4.07	1.61	5.47	0.42	20.53	2.92
	**Moneymaker**	5.04	1.42	0.33	3.81	0.88	7.13	0.85	45.67	4.83
	**Cherry-INRAE (2)**	1.33	1.20	1.35	4.20	1.73	4.33	1.76	17.53	4.85
	**Seccagno PSC1-1**	4.81	0.60	0.62	0.52	1.06	5.27	0.78	35.50	9.65
	**tomataki**	2.03	1.36	0.15	6.48	0.48	1.73	0.83	32.16	0.24
	**CC_1791 Allungato a Fiasco**	3.53	0.60	0.53	0.72	1.23	4.89	0.24	28.11	7.81
	**CC_1665 Pollena**	3.06	0.17	1.00	2.23	0.39	3.35	0.56	7.06	6.66
	**GR-451/04**	2.89	0.07	1.00	1.82	0.54	4.30	0.76	23.99	1.82
	**Corbarino**	1.53	0.88	0.76	2.91	1.53	4.92	0.67	18.11	3.74
	**Cherry-INRAE (1)**	6.01	0.76	0.28	3.67	0.54	7.87	2.42	12.49	3.56
	**Cherry-INRAE (3)**	1.45	0.03	0.59	3.13	0.30	2.47	0.16	13.01	2.09
	**Cherry-INRAE (4)**	1.00	0.17	0.87	4.02	1.10	3.36	0.89	7.85	4.51

Table S3. Standard errors of means (n =3) of the concentration of macro- (K, Na, Ca, Mg and K/Na) and micro- nutrients (Fe, Cu, Mn, Zn) in fruit of different tomato landraces.

		Fruit								
**Salinity Stress**	**Variety**	**K (mg/g)**	**Na (mg/g)**	**K/Na**	**Ca (mg/g)**	**Mg (mg/g)**	**Fe (μg/g)**	**Cu (μg/g)**	**Mn (μg/g)**	**Zn (μg/g)**
		**Interraction**								
**0.5 mM NaCl**	**Chondrokatsari**	0.65	0.07	10.94	0.02	0.07	3.24	0.34	0.20	0.99
	**Valldemossa (de)**	2.12	0.12	6.14	0.00	0.09	5.39	0.18	0.86	0.99
	**Areti**	1.08	0.03	5.91	0.02	0.08	4.71	0.43	0.36	0.60
	**ATS-048/06**	0.63	0.05	5.36	0.01	0.06	1.80	0.11	0.55	0.88
	**de Ramellet**	1.31	0.04	3.33	0.00	0.07	6.61	0.73	0.72	2.95
	**Moneymaker**	2.08	0.05	6.78	0.00	0.08	1.57	0.47	0.32	1.05
	**Cherry-INRAE (2)**	0.82	0.07	7.84	0.01	0.08	3.33	0.25	1.39	0.91
	**Seccagno PSC1-1**	1.39	0.03	3.26	0.04	0.06	2.90	0.43	0.28	0.98
	**tomataki**	2.14	0.03	1.66	0.02	0.06	1.25	0.53	0.65	1.10
	**CC_1791 Allungato a Fiasco**	0.75	0.01	1.03	0.00	0.05	1.73	0.34	0.49	0.84
	**CC_1665 Pollena**	1.78	0.07	12.36	0.01	0.07	3.07	0.74	0.41	0.91
	**GR-451/04**	1.03	0.04	9.26	0.02	0.06	5.14	0.40	0.91	1.35
	**Corbarino**	1.25	0.05	12.27	0.00	0.06	3.57	0.54	0.28	0.41
	**Cherry-INRAE (1)**	0.95	0.03	7.99	0.02	0.09	1.47	0.33	0.31	0.80
	**Cherry-INRAE (3)**	0.75	0.17	15.90	0.02	0.03	1.21	0.60	0.35	1.72
	**Cherry-INRAE (4)**	1.20	0.04	9.55	0.02	0.09	3.14	0.54	0.50	0.64

30 mM NaCl	**Chondrokatsari**	1.31	0.13	1.29	0.02	0.06	7.44	0.62	0.48	1.36
	**Valldemossa (de)**	0.41	0.11	2.59	0.00	0.02	2.55	0.37	0.53	1.73
	**Areti**	0.85	0.11	2.72	0.01	0.07	2.66	0.19	0.55	0.94
	**ATS-048/06**	2.61	0.11	2.65	0.02	0.10	3.45	0.43	0.64	1.37
	**de Ramellet**	0.75	0.10	2.37	0.00	0.03	6.17	0.49	0.54	1.94
	**Moneymaker**	1.44	0.13	2.12	0.00	0.06	0.86	0.69	0.67	1.35
	**Cherry-INRAE (2)**	2.89	0.09	1.10	0.01	0.05	2.28	0.51	0.62	1.08
	**Seccagno PSC1-1**	1.60	0.09	1.79	0.01	0.05	2.84	0.32	0.27	1.31
	**tomataki**	1.35	0.07	0.92	0.01	0.04	3.85	0.24	0.51	1.08
	**CC_1791 Allungato a Fiasco**	0.71	0.10	4.00	0.03	0.04	1.95	0.33	0.39	0.74
	**CC_1665 Pollena**	0.71	0.02	1.51	0.00	0.03	1.20	0.47	0.13	0.21
	**GR-451/04**	1.80	0.22	10.02	0.02	0.05	1.95	0.47	0.39	0.94
	**Corbarino**	0.75	0.09	6.58	0.02	0.04	3.34	0.51	0.36	0.99
	**Cherry-INRAE (1)**	0.95	0.03	2.17	0.01	0.02	2.52	0.19	0.24	0.63
	**Cherry-INRAE (3)**	0.41	0.04	1.18	0.02	0.03	1.97	0.26	0.31	0.16
	**Cherry-INRAE (4)**	1.20	0.04	3.10	0.01	0.04	1.85	0.31	0.37	0.28
